# Building and Sustaining Flu Vaccine Acceptance and Trust in the Black Community through Partnerships with Churches, Salons, and Barbershops

**DOI:** 10.1007/s40615-024-02141-7

**Published:** 2024-09-06

**Authors:** Henry Nuss, Lois Privor-Dumm, Chinonso Ukachukwu, Laura Lee Hall

**Affiliations:** 1 New Orleans School of Public Health, LSU Health, Behavioral Community Health Sciences, New Orleans, LA USA; 2 Johns Hopkins Bloomberg School of Public Health, International Vaccine Access Center, Baltimore, MD USA; 3 Center for Sustainable Health Care Quality and Equity, National Minority Quality Forum, Washington, DC USA

**Keywords:** Influenza vaccination, Racial disparities, Barriers to immunization, Community leaders, Faith-based, Trust

## Abstract

Despite the benefits of flu vaccines, Black adults continue to experience lower vaccination rates in the United States. Contributing factors include lack of access to health care and trusted information about vaccines. The National Minority Quality Forum’s Center for Sustainable Health Care Quality and Equity collaborated with church pastors, barbers, and hair stylists to disseminate a survey to their communities to assess barriers/facilitators to flu vaccine uptake. The population (*n* = 262) was mostly Black (93%), female (77%), between the age of 50–64 (39%) and vaccinated (73%). The most common reasons cited by respondents for being vaccinated were personal health, a habit of getting the shot, and a desire not to spread it to others. Among the unvaccinated (27%), the most common reasons for not vaccinating were lack of perceived need, concern the shot would make them sick, and that they do not normally get vaccinated. Vaccine knowledge and trust in health care providers’ recommendations was higher amongst vaccinated individuals. Amongst the unvaccinated, trust was lower and there was a stronger belief that the vaccine would not prevent illness. Age was also associated with the likelihood of being vaccinated and greater vaccine knowledge and trust in provider recommendations. Unvaccinated individuals, particularly those under 54 years of age, did not hold strong distrust, attitudes were more neutral, and concern for others was moderate, suggesting an opportunity to target younger age groups. This study highlights the importance of trusted community messengers in conveying targeted messages on the safety and effectiveness of the flu vaccine.

**Supplementary Information:**

The online version contains supplementary material available at 10.1007/s40615-024-02141-7.

## Introduction

Black communities in the United States (U.S.) face a disproportionate burden of disease, putting individuals at increased risk for hospitalizations or premature death. Decreased vaccination rates for communicable diseases such as influenza (flu) are among the disparities experienced by Black individuals. Despite strong evidence that flu vaccines help prevent some of the most severe health outcomes, Black adults in the U.S. were less likely to be vaccinated for flu than White adults during the 2022–2023 flu season (39.3% vs. 52.8) [1]. As a result, Black adults are hospitalized for flu-related complications nearly twice as often as White adults [2]. One study of Medicare beneficiaries showed even further disparities in coverage with only a third of Black beneficiaries receiving a seasonal flu vaccine compared to nearly half of White beneficiaries. Further, of those over the age of 65, one-third of Black beneficiaries received the recommended high-dose vaccine as compared to White individuals, reflecting structural racism [3, 4].

The reasons for disparities in flu vaccination are multiple and have been attributed to issues of access to both health care and insurance, higher rates of underlying health conditions, missed opportunities to vaccinate, misinformation about vaccines, and mistrust of the health system [5]. The historical distrust of the health system amongst the Black community is well noted [6, 7]. Subsequently, this lack of trust has been a major factor in Black adults choosing not to be vaccinated, resulting in health outcome disparities [8, 9]. Additionally, there is a greater belief in myths and misconceptions regarding vaccines among Black adults [8, 10]. A greater portion of Black individuals do not trust the health system or have had a bad experience with their provider as compared to White individuals [11]. Health providers and trusted sources are an important influence in vaccination, thus reducing the likelihood of vaccination when trusted sources are not providing nudges [12]. Therefore, to reduce disparities, listening to the community and working with trusted intermediaries when providing information have shown promise [13–16].

**Fig. 1:**
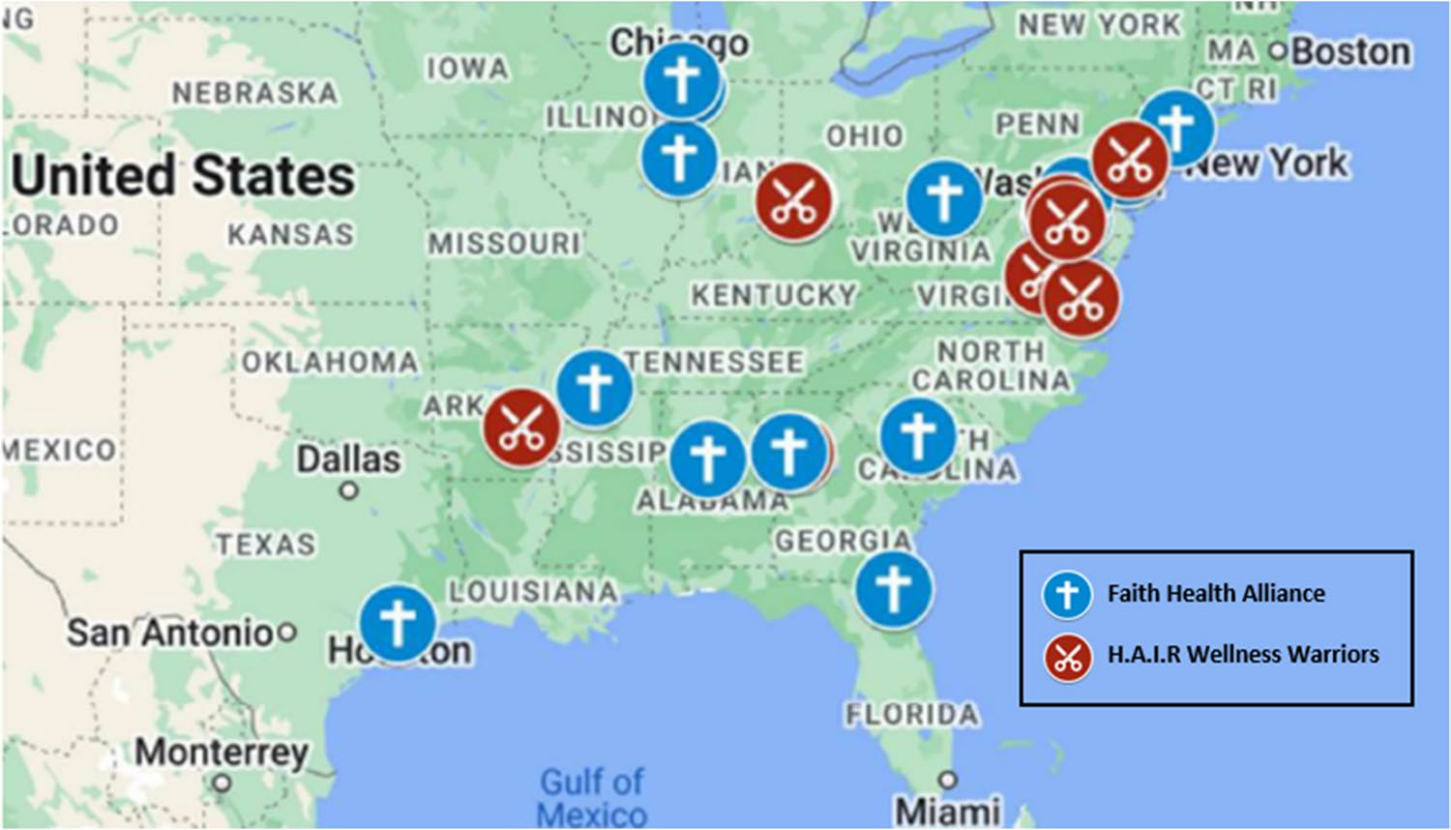
Center for Sustainable Health Care Quality and Equity Faith Health Alliance and Health Advocates In-Reach and Research (HAIR) Wellness Warriors

A previous successful pilot program in Baltimore, Maryland, promoting flu vaccinations [5] and significant literature pointing to the agency of churches [17], and barbers and stylists’ role in health promotion in underserved communities [18] led the Center for Sustainable Health Care Quality and Equity (SHC) to support national health promotion efforts in predominantly Black communities beginning in 2021. This included a national network of church leaders– the Faith Health Alliance (FHA) – and barbers and hair stylists – Health Advocates In-Reach and Research (HAIR) Wellness Warriors - to promote vaccine equity, including 20 churches and ten HAIR Wellness Warriors (Fig. 1). Recruitment for the program was conducted in collaboration with FHA co-chairs and the University of Maryland’s Center for Health Equity and was informed by geo-mapping analysis of both flu vaccine disparities and demographic profiles across the US [19]. Churches, barbers, and stylists received an honorarium to support their activities during program participation and SHC staff worked with expert partners from Johns Hopkins University International Vaccine Access Center (JHU/IVAC) and the University of Maryland (UMD) to train and support the community leaders, with regular conference calls hosted for the two groups to facilitate peer-to-peer learning. Community leaders hosted several activities including educational programs, health fairs, and onsite vaccination. An average of 1,000 people were reached through the activities. To assess the impact of these programs, a survey was developed and implemented to assess the knowledge, beliefs, and actions concerning influenza vaccination of program participants. This paper summarizes the vaccine status and associated viewpoints of influenza vaccination overall and by gender and age group in the communities surveyed during the 2022-23 flu season to get an early indication of the prevalent attitudes, identify gaps, and guide future activities.

## Methods

### Survey

After a review of previous survey instruments, program staff, community stakeholders, and program evaluation experts created a survey on Google Forms to reveal the characteristics of flu vaccine program participants in FHA and HAIR Wellness Warriors organizations and to probe knowledge, beliefs, and practices related to flu vaccination [8, 20]. The survey included ten questions about demographics (age groups, gender, race/ethnicity), vaccination status, multiple-choice questions for reasons for vaccination or non-vaccination as well as questions using a five-point Likert scale to assess facilitators and barriers of flu vaccination. (Supplementary Information). After the initial draft of the survey was created by content experts for face validity and alignment with previously used surveys, it was reviewed by selected community members (participants of the program) and health professionals for content and cultural appropriateness. Modifications to the survey were made based on feedback and revised by content experts for overall fit and clarity. The link to the live survey was then delivered in February of 2023 to pastors and HAIR Wellness Warriors who distributed it among their congregants and patrons. The survey remained open for three weeks. Responses were anonymous, checked for accuracy and completeness, and then transferred to a secure dataset for analysis. The survey protocol was reviewed by the WCG Clinical Services Institutional Review Board and deemed exempt 45 CFR § 46.104(d)(2).

### Statistical Analysis

All statistical analyses were conducted using IBM SPSS software (version 26). Descriptive statistics were used to summarize the frequency and percentages for the demographic variables, vaccination status, and responses to survey questions/items. Unadjusted vaccination rates by demographic variables were calculated. Cross tabulations were used to compare the distribution of responses according to various respondent characteristics (e.g., vaccination status, age) and significance was tested using the Chi-square statistic.

## Results

### Demographics

We received 290 complete surveys. Twenty-eight surveys were eliminated from the analysis due to missing vaccination status or responders who indicated that they were both vaccinated and unvaccinated. For the remaining sample (*n* = 262), the majority of respondents were Black and female between the age of 50 to 64 (Table 1).

**Table 1:** Demographics of survey sample (*N* = 262)

	*n*	*%*
Gender		
Female	203	77.5
Male	59	22.5
Age (range)		
18–49	72	27.5
50–64	101	38.5
65+	81	30.9
Prefer not to say	8	3.1
Race/Ethnicity		
Black	244	93.1
White	9	3.4
More than one	5	1.9
Prefer not to say	3	1.1
Hawaiian/Pacific Islander	1	0.4
Vaccination status		
Vaccinated	191	72.9
Not vaccinated	71	27.1

### Vaccination status

Approximately 73% (*n* = 191) of the sample indicated on their surveys that they were vaccinated for flu. Women were no more likely to be vaccinated or unvaccinated than men, χ² (2, *N* = 262) = 0.80, *p* = 0.67 (Table 2). Individuals in the 65 + age group were more likely to be vaccinated than those in the younger age groups, χ² (3, *N* = 262) = 14.7, *p* = 0.002.

**Table 2:** Vaccination status by gender and age groups

	Vaccinated		Unvaccinated		*p-*value
	* n *	%	* n *	%	
Gender					0.67
Female	149	73.8	53	26.2	
Male	41	69.5	18	30.5	
Age Groups					0.002
18–49	40	61.1	28	38.9	
50–64	70	69.3	31	30.7	
65+	71	87.7	10	12.3	

### Reasons for being vaccinated

The top three reasons listed for becoming vaccinated were “my personal health” (*n* = 138, 72.3%%), followed by “I always get my flu shot every year” (*n* = 89, 46.6%), and “don’t want to spread it” (*n* = 59 m 30.9%), respectively (Table 3). When compared by gender, women reported these same top three reasons for being vaccinated. Among men, the top third reason was the convenience of receiving the vaccine (26.8% vs. 22.8%, ns). When compared by age groups, the 50–64 age group differed slightly in their top three reasons in that they reported they were vaccinated because their doctor said they should as the third most common reason (not statistically significant).

**Table 3:** Top three reasons why vaccinated participants got vaccinated, all and by age groups

	Reason (ranked)	Frequency	%
All (191)	1) My personal health	138	72.3
	2) I always get my shot every year	89	46.6
	3) Don’t want to spread it	59	30.9
Age Groups			
18–49 (*N* = 44)	1) My personal health	34	77.3
	2) I always get my shot every year	14	31.8
	3) Don’t want to spread it	13	29.5
50–64 (*N* = 70)	1) My personal health	45	64.3
	2) I always get my shot every year	27	38.6
	3) Doctor says I should	21	30.0
65+ (*N* = 71)	1) My personal health	56	78.9
	2) I always get my shot every year	45	63.4
	3) Don’t want to spread it	29	40.8

### Reasons for being unvaccinated

For all participants who reported that they were unvaccinated (*N* = 71), the most common reason cited for not receiving the vaccine was “I don’t think I need it” (*n* = 30, 42.3%), followed by “I’m worried it might give me the flu” (*n* = 25, 35.2%) and then “I just don’t get flu shots” (*n* = 8, 11.3%) (Table 4). When broken down by age groups, the top reason for each age group was “I don’t think I need it”, followed by “I’m worried it might give me the flu.” Some differences were observed for the third most common reason for not being vaccinated by age group. For example, one of the four respondents in the 18–25 age group said cost was a factor. In comparison, three of the 24 respondents in the 26–49 age group said they did not think it was safe. For the two older age groups, respondents said that they simply never get the flu vaccine as the third most common reason.

**Table 4:** Top three reasons why unvaccinated participants were not vaccinated, all and by age groups

	Reason (ranked)	Frequency	%
All (*n* = 69)	1) I don’t think I need it	30	42.0
	2) I’m worried it might give me the flu	25	36.2
	3) I never get flu shots	8	11.6
Age groups			
18–49 (*n* = 28)	1) I don’t think I need it	17	60.7
	2) I’m worried it might give me the flu	10	35.7
	3) Don’t think it’s safe	3	10.7
50–64 (*n* = 31)	1) I don’t think I need it	10	32.3
	2) I’m worried it might give me the flu	12	38.7
	3) I never get flu shots	5	16.1
65+ (*n* = 10)	1) I don’t think I need it	2	20.0
	2) I’m worried it might give me the flu	2	20.0
	3) I never get flu shots	2	20.0

### Flu vaccine beliefs and attitudes

We proposed five statements regarding various aspects of the flu vaccine to ascertain attitudes and beliefs towards vaccine acceptance. For the entire sample, 64% agreed with the statement, “The flu shot can prevent me from getting sick and/or reduce how sick I get if I do get it.” Of those who agreed, 90.4% were vaccinated and 9.6% were unvaccinated (Table 5). 87% of those who agreed that it is never too late in the flu season (August through February) were vaccinated compared to 13% who were unvaccinated. Twenty-seven respondents agreed with the statement, “It’s not safe to get the flu shot.” Of those, 63% were vaccinated, although that percentage was not statistically significant. Approximately two-thirds of all respondents said they trust their health care providers when advised to receive the flu vaccine, the majority of which were also vaccinated (151 out of 169). Lastly, 86% of all respondents said they knew a place nearby where they could obtain a flu shot and vaccinated responders were more likely than unvaccinated responders to possess that information.

**Table 5:** Responses to survey statements by vaccinated and unvaccinated groups. Likert-type response options were “strongly disagree” (1) to “strongly agree” (5). The most frequent response option given for each statement is in bold type

	Vaccination status groups									
	Vaccinated (*n* = 190)					Unvaccinated (*n* = 71)				
Survey statements	1 Strongly disagree	2	3	4	5 Strongly agree	1 Strongly disagree	2	3	4	5 Strongly agree
The flu shot can prevent me from getting sick and/or reduce how sick I get if I do get it.	12 (6)	6 (3)	21 (11)	25 (13)	123 (66)*	7 (9.9)	15 (21)	**33 (47)**	9 (13)	7 (10)
It’s never too late in the flu season (Aug-Feb) to get the vaccine	13 (7)	10 (5)	32 (17)	21 (11)	113 (59)*	6 (9)	11 (16)	**33 (47)**	8 (11)	12 (17)
It’s not safe to get the flu shot.	145 (76)*	21 (11)	8 (4)	4 (2)	13 (7)	13 (18)	16 (23)	**32 (45)**	6 (9)	4 (6)
I TRUST health care providers when they say I should get the shot.	11 (6)	8 (4)	21 (11)	32 (17)	119 (62)*	7 (10)	14 (20)	**32 (45)**	13 (14)	8 (11)
I know a place near me where I can get a flu shot.	9 (5)	4 (2)	5 (3)	12 (6)	160 (84)*	5 (7)	2 (3)	6 (9)	11 (16)	**43 (60)**

*Vaccinated response distributions were significantly different from unvaccinated responses for all survey items, *p* < 0.01

### Flu vaccine beliefs and attitudes between age groups

When compared by age, the 65 + age group was more likely to agree with the statement, “The flu shot can prevent me from getting sick and/or reduce how sick I do get if I get it” (*p* = 0.02) and “It’s never too late in the flu season to get the vaccine” (*p* = 0.03) than the younger age groups (Table 6). Participants who were aged 50 or older were also more likely to agree with the statement “I trust my health care providers when they say I should get a shot” than the 18–49 age group (*p* = 0.001).

**Table 6:** Responses to statements about the flu vaccine by age groups. 1 = strongly disagree, 5 = strongly agree with chi-square statistic

	Response options	Age groups			χ²
		18–49	50–64	65+	
Survey item		n(%)	n(%)	n(%)	
The flu shot can prevent me from getting sick and/or reduce how sick I get if I do get it.	1	4 (6)	6 (6)	9 (11)	(12, 261) = 30.6, *p* = 0.002
	2	7 (10)	7 (7)	7 (9)	
	3	**25 (35)**	22 (22)	5 (6)	
	4	12 (17)	15 (15)	6 (7)	
	5	24 (33)	**50 (50)**	**54 (67)**	
It’s never too late in the flu season (Aug-Feb) to get the vaccine.	1	4 (6)	4 (4)	11 (14)	(12, 259) = 29.3, *p* = 0.004
	2	6 (8)	9 (9)	6 (7)	
	3	25 (35)	32 (32)	6 (7)	
	4	7 (10)	13 (13)	9 (11)	
	5	**31 (42)**	**40 (40)**	**46 (61)**	
It’s not safe to get the flu shot.	1	**34 (47)**	**55 (55)**	**65 (80)**	(12, 262) = 31.6, *p* = 0.002
	2	18 (25)	13 (13)	5 (6)	
	3	16 (22)	18 (18)	5 (6)	
	4	3 (3)	5 (5)	2 (3)	
	5	3 (3)	10 (10)	4 (5)	
I trust health care providers when they say I should get the shot.	1	3 (4)	7 (7)	8 (10)	(12, 262) = 33.5, *p* = 0.001
	2	9 (13)	9(9)	4 (5)	
	3	**26 (36)**	19 (19)	7 (9)	
	4	12 (17)	20 (20)	9 (11)	
	5	22 (31)	**46 (46)**	**53 (65)**	
I know a place near me where I can get a flu shot.	1	1 (1)	6 (6)	7 (9)	(12, 257) = 9.3, *p* = 0.68
	2	1 (1)	3 (3)	2 (3)	
	3	4 (6)	4 (4)	2 (3)	
	4	7 (10)	11 (11)	5 (6)	
	5	**57 (79)**	**75 (74)**	**64 (79)**	

## Discussion

The overall vaccination rate for this survey sample was relatively high at 72.9%, as compared to a March 2023 CDC report which cited 39.3% of Black adults surveyed in the National Immunization Survey Adult COVID Module (NIS-ACM) [21]. The Behavioral Risk Factor Surveillance System (BRFSS) for the 2021-22 influenza season showed 42.0% coverage of Black adults vs. 53.9% of White adults, were vaccinated, regardless of insurance coverage, having a health care provider, or a medical checkup in the past year [2]. While different methodologies typically yield different results, the higher rates among the program participants in the present survey suggest either a positive impact of the community-based programs or a more highly motivated group of individuals, or both [1, 22].

In our sample, age was associated with vaccination status. Specifically, older individuals were more likely to be vaccinated. Other reports have found similar results [23, 24]. Reasons for this occurrence may be that older adults are in more frequent contact with a health care provider or become more health conscious and/or knowledgeable with age. It is noteworthy that the older adults (age 65+) in our sample had a higher vaccination rate (88%) than a comparable national sample (71%) from the same CDC data noted above (71.0%) [1, 2]. Similarly, vaccination rates were higher in the younger age groups (e.g., less than 65 years of age) than nationally reported data of Black individuals [21]. These findings suggest a positive influence of the programs, which included educational presentations and print materials as well as vaccine clinics, as assessed post-intervention.

Among those who were vaccinated, the most frequently cited reason for being vaccinated was to protect personal health. The vast majority (80%) of vaccinated participants also believed that the flu vaccine was effective in preventing infection or abating the severity of illness if they were to become infected, consistent with findings in other studies [25]. We found that people who chose to be vaccinated for health reasons also had strong beliefs that the vaccine would be beneficial. We also observed that individuals who had received the flu vaccine also understood that it is never too late to receive a vaccine during flu season. These data suggest the importance of knowledge of the effectiveness of the flu vaccine as one factor in terms of promoting vaccine acceptance [2, 24, 25].

Approximately 47% of vaccinated participants said that they “always” get their shot as the second most common reason for being vaccinated which is linked to increased knowledge of flu vaccine effectiveness in previous studies [26]. It is not clear whether the remaining 53% of vaccinated responders who did not indicate that they always received the vaccine got the shot every flu season. If this is indeed the case, it is possible that these individuals are vaccine friendly, but may need additional incentives or motivation to make it routine. For example, a previous qualitative study of patients with diabetes found that a stable habit of flu vaccination was supported by providing coupons for free vaccines and regular doctor visits contributing to the routine [27]. Those who routinely get a flu vaccine also are more likely to access vaccinations in various settings [28], suggesting that inconsistent vaccinators require more options for venues to obtain the vaccine. Another study showed that past flu vaccination was the strongest predictor of vaccination [29]. The programmatic implications include nurturing routine vaccination through annual reminders, education, and access support. It is notable that a previous study also observed that a history of non-vaccination is also predictive, which we observed as one of the top reasons in our unvaccinated group [8].

The third leading reason for receiving the flu shot was a desire not to spread the flu, indicated by 30.9% of all respondents and 40.8% of the oldest group. As found in other studies of vaccine acceptance, altruism was a top reason for vaccine acceptance [30]. Quinn concluded that:

*“Our research on the influence of family and social networks on vaccine behavior*,* coupled with the CDC’s findings that messages focused on the collective good and protecting one’s family are effective*,* reinforce the critical importance of reaching African American families and communities. Certainly*,* HCP can reinforce the potential impact of flu vaccination in protecting other members of families. Equally vital is the need to engage community organizations including faith communities*,* trusted opinion leaders for civic groups*,* and large organizations such as sororities*,* fraternities*,* and others to promote the importance of flu vaccination as a means to protect the broader community”* [30].

Unvaccinated respondents reported the number one reason for not getting the flu vaccine was the view that they do not need it, including the majority of adults under 50 years of age. Other studies show this belief is common among other unvaccinated populations, citing that they do not believe the flu will make them very sick or that the recommendations do not apply to them [25]. This is of concern, given the higher risk of serious illness and hospitalization in the Black community [2] and the association of poor flu outcomes among people with various chronic conditions such as heart disease, diabetes, and chronic kidney disease [31–33], which are much more prevalent in the U.S. Black population at a younger age and often undiagnosed.

The unvaccinated survey respondents as well as the youngest group overall had less knowledge about the flu vaccine, in terms of its ability to help prevent serious illness, timing of flu vaccination, and vaccine safety. In addition, these two groups were less likely to trust health care provider recommendations. While education alone is unlikely to increase vaccine acceptance, the Community Preventive Services Task Force (CPSTF) has concluded that education in combination with clinic and community-based strategies is effective [34]. More targeted communications in terms of content (e.g., risk, safety, effectiveness, relevance in chronic disease), cultural relevance as well as the platform by which it is delivered, are needed [25].

The role of the health care provider is essential, as a strong recommendation and provision of the vaccine are highly associated with increased vaccination rates, but are often lacking. However, if people of color who are unvaccinated lack trust in health care providers, their recommendation may be less effective [35]. This may in part be due to how Black individuals are treated or perceived to be treated, whether the influenza vaccine is discussed or offered, or the message itself [35]. Furthermore, structural racism, which contributes to a lack of trust, has also been shown to be a contributing factor of COVID-19 vaccine hesitancy among Black adults [36]. Although we did not include structural racism in our survey, it is likely also to play a role on flu vaccine acquisition and should be considered when discussing the benefits of the flu vaccine with similar audiences.

Community engagement affords health care providers an opportunity to better understand the culture of individuals in their communities and practices and to build trust, including among family and friends that constitute the social fabric [37]. Engagement with the community, including through the provision of education and vaccines in settings such as churches, salons, and barbershops, can strengthen the provider’s role as a trusted source of information and services for many Black people [38].

Beyond lack of knowledge and trust, structural inequities, such as lack of insurance, transportation or inability to pay, have been shown to underpin the lack of flu vaccination [39]. A systematic review for H1N1 looked at factors influencing vaccination and notes that access and convenience should be prioritized in Black communities [40]. We did not specifically include this question in our survey, but hypothesize Black churches, community centers and barbershops and salons are all places where access can be facilitated. Ensuring that programs, if not offering vaccines themselves, have information on where vaccines can be given free of cost for both those with or without insurance is essential. Nearly all of the people in our sample indicated that they knew where to get a vaccine, but focusing on providing that information to the unvaccinated is still needed. Further research is likely needed on the messages that work in the Black community to help reduce disparities [41].

Some responses in our study among the unvaccinated demonstrate what likely does not resonate especially for young Black individuals, namely that the vaccine will prevent them from getting sick. One study showed that being young, male, Black and/or without insurance resulted in significantly lower odds of getting a seasonal flu vaccine, suggesting that this is a group to target for messaging [42]. Just under 23% of unvaccinated people (largely younger individuals) believe the vaccine will not keep them from getting them sick, but many are still neutral on this belief, indicating an opportunity to change opinion and particularly provide information on the potential severity of disease not only for them, but those at higher risk around them that may have underlying conditions. Building expectations around the flu and prevention of severe illness or death may help address the finding that they do not think they need the vaccine. It may not be for themselves; it may be based on the idea they can spread it to others.

Trust is another major difference between those that have gotten the vaccine and those that have not. Only one fourth of the sample of unvaccinated people trusted their health provider’s opinion when they recommended a flu shot, especially among the younger respondents, while 79% of those that did get the vaccine were trusting of the health provider’s recommendation. The implications are that messages should be targeted and tailored to younger individuals in addition to continued messaging to older individuals. Health care providers also will need support with addressing mistrust among younger Black patients. Engaging Black providers is also a recognized strategy to ensure trust in underserved communities and efforts to engage those providers need to be part of any equity strategy [43].

Within the groups of trusted messengers, it is important to foster culturally appropriate dialogue to build trust as well as a patient-centered approach [13, 15, 30, 37]. Churches, barbers and salons have a special place in the community, and they have been engaged in both influenza and COVID-19 campaigns [15, 17, 25, 44, 45].

Coordinating with government institutions and health systems is important as they play an important role in providing accurate information, not only to the public but to trusted messengers [46]. Effective, sustained, and mutually accountable partnerships among community organizations, health systems, and health departments are essential in building trust [47, 48].

Finally, in addition to trust, appropriately focused education is key. A tailored approach to communication grounded in the principles of behavior change [49, 50] is what churches, barbershops and salons do best, offering a safe space for authentic conversations, and actions to boost physical, mental, and/or spiritual well-being [51]. This means developing an education program that: provides essential information on flu and vaccine safety and effectiveness; discusses the risk and burden of disease *specific* to the community; talks about the benefits and the risks of flu and vaccines; and addresses rumors and myths in a respectful manner. Community-based organizations like those engaged in this program are well placed to tailor messages to address the cultural norms and lifestyle of the people they serve, including family networks. For example, messaging that addresses the protection of others in a given community, preventing hospitalization, missed work or school days, or simply enabling visits with older and vulnerable family members, may persuade unvaccinated individuals. These findings suggest a tailored message to each age group to address their specific concerns or barriers when communicating with those individuals.

## Limitations

There are some limitations to this study, including selection bias of people who were vaccinated and likely a disproportionate number of healthy individuals [52]. To address this issue in the future, we could be more deliberate about reaching unvaccinated individuals. Second, the survey did not differentiate individuals who received the flu vaccine every year versus those who received the flu vaccine occasionally, preventing us from learning why some people did not consistently get the vaccine. We did not conduct a pilot test of the final draft of the survey. However, we did have the final draft reviewed by members of the target community, content and evaluation experts to address content validity. Further, we did not collect information on certain participant demographics, such as insurance status, income, and other social determinants of health that could have provided a more detailed explanation of the results we observed and improved the generalizability of our findings. We should also note that 28% of our sample was under the age of 49. To this point, other researchers and health care providers should consider this statistic when comparing to their own populations which may have different age distributions.

## Conclusions

This study highlights the importance of a trusted community messenger. For people who trust their health provider, the church, salon, or barbershop leader can reinforce knowledge and behavior while providing additional messaging suited to the culture and concerns of their community. For individuals who do not trust health care providers, community champions can be an important bridge between the Black community and providers and public health experts, building an environment of trust and engagement, particularly for younger age groups. Engaging churches, barbers and stylists to build and sustain flu vaccine acceptance through education and connection to health care provides a promising approach to reduce disparities in flu vaccination rates between African Americans and Whites.

## Supplementary Information

Below is the link to the electronic supplementary material.

ESM 1 (DOCX 16.5 KB)
