# Supplementary material for: Building and Sustaining Flu Vaccine Acceptance and Trust in the Black Community through Partnerships with Churches, Salons, and Barbershops
Source: J Racial Ethn Health Disparities. 2024 Sep 6;12(5):3365–74. doi: 10.1007/s40615-024-02141-7 (PMC12446130; doi:10.1007/s40615-024-02141-7)
Supplement: Supplementary file 1 — (DOCX 16.5 KB) [file 40615_2024_2141_MOESM1_ESM.docx]

Hello. Thank you for agreeing to allow us to resubmit with revisions. We have made the following changes per Reviewer 4’s comments as shown below. We hope these changes are sufficient to allow this manuscript to move forward to publication. Please let us know if there are any further revisions necessary after reviewing these changes.

**Reviewer 4 comment #1:** *“Is there a reason why "Churches" is capitalized throughout the manuscript? As a common noun, this should be lowercase unless at the beginning of a sentence, similar to how barber and stylist is referenced throughout.”*

Response to comment #1: We thank you for your comment. We have changed “churches” to lower case per your recommendation in the following locations on the manuscript:

- Page 4, lns 9 and 12
- Page 12, ln 20
- Page 15, ln 5
- Page 16, lns 8, 13 and 18

**Reviewer 4 comment #2: Results/Limitations** - *The survey demographics are quite limited. Having limited demographics should be listed as a limitation as understanding how insurance status, SES, housing situations, and other social determinants of health affect or influence flu vaccination could have provided more nuanced insight/associations into your analysis and results.*

Response to comment #2: We agree with this comment and have added comments related to this in the limitations section (see page 16, lns 4-7.)

**Reviewer 4 comment #3: Results/Limitations** - *Only 28% of the sample were < 49 years old, so these results may not be generalizable to all Black individuals, church, barbershop and salon goers and should be added to the limitations section.*

Response to comment #3: Thank you for your comment and keen observation. We added verbiage to address this concern (see page 16, lns 8 – 10).

Other changes made not in response to reviewer comments:

1. Page 4, ln 14 – replaced “in order to” to “to”
2. Page 5, ln 2 – replaced “In order to” to “To”
   - 1 and 2 were done to eliminate redundancy of phrasing.
3. Page 9, ln 13 – fixed a typo (suggestsa to suggest a)
